# Objective Observer vs. Patient Motor State Assessments Using the PD Home Diary in Advanced Parkinson's Disease

**DOI:** 10.3389/fneur.2022.935664

**Published:** 2022-07-12

**Authors:** Jonathan Timpka, Matthias Löhle, Alexander Bremer, Sofia Christiansson, Florin Gandor, Georg Ebersbach, Örjan Dahlström, Susanne Iwarsson, Maria H. Nilsson, Alexander Storch, Per Odin

**Affiliations:** ^1^Division of Neurology, Department of Clinical Sciences Lund, Lund University, Lund, Sweden; ^2^Department of Neurology, Skåne University Hospital, Lund, Sweden; ^3^Department of Neurology, University of Rostock, Rostock, Germany; ^4^German Center for Neurodegenerative Diseases (DZNE) Rostock/Greifswald, Rostock, Germany; ^5^Movement Disorders Clinic, Beelitz-Heilstätten, Germany; ^6^Department of Neurology, Otto-von-Guericke University, Magdeburg, Germany; ^7^Department of Behavioural Sciences and Learning, Linköping University, Linköping, Sweden; ^8^Athletics Research Center, Linköping University, Linköping, Sweden; ^9^Department of Health Sciences, Lund University, Lund, Sweden; ^10^Memory Clinic, Skåne University Hospital, Malmö, Sweden

**Keywords:** motor fluctuations, Parkinson disease, patient reported outcome (PRO) measures, clinical trials, Parkinson disease home diary

## Abstract

**Background:**

The Parkinson Disease (PD) Home Diary (HD) is a commonly used clinical outcome measure, but it has not been extensively compared to direct assessments by experienced observers.

**Objective:**

Validation of patient-reported HD by investigating the agreement between motor state assessments by patients and observers.

**Methods:**

This observational study included patients with PD and motor fluctuations. Observers were physicians or research nurses. Patients completed a screening visit, one day of diary ratings at home, and then two days of ratings on-site during which patients and observers simultaneously judged the participants' motor state.

**Results:**

Observers and 40 patients completed 1,288 pairs of half-hourly blinded motor state assessments. There were significant differences between observer and patient ratings (*P* < 0.001) and the temporal agreement was poor (Cohen's κ = 0.358). The agreement between patient and observer ratings was 71.1% for observed “On without dyskinesia”, 57.3% for observed “Off”, and 49.4% for observed “On with dyskinesia”. Daily times spent in the three motor states as aggregated diary data showed fair to excellent reliability with intraclass coefficient values ranging from 0.45 to 0.52 for “On” and 0.77 for “Off”.

**Conclusion:**

There were significant differences between observer and patient ratings. Patients and observers generally agreed on when the patients was in the “On” state (with or without dyskinesia). Patient ratings on the hour level seem to be influenced by other aspects of the patients' experience than the observed motor state, but assessment of daily time spent in the different motor state provides reasonable reliability.

## Introduction

In the early 2000's Hauser et al. developed the Parkinson Disease (PD) Home Diary (HD), or “Hauser diary”, for use as an outcome measure of motor function in clinical trials (1–3). Previous to the HD, trials relied on the reduction of time spent in “off” as an indicator of improved motor function and did not address any potential increase of dyskinesia. In addition to “On” and “Off”, Hauser et al. added “On with non-troublesome dyskinesia” and “On with troublesome dyskinesia” to better reflect the patient's motor state.

The HD was validated through correlation between patient self-assessment of “On” or “On without troublesome dyskinesia” with “good” time, then “Off” or “On with troublesome dyskinesia” with “bad” time (2). The predictive validity was reasonable when testing the correlation between HD ratings and patients' responses to questions about their motor state and the HD subsequently showed a good test-retest reliability (1). Patients often have limited knowledge of motor state terminology and may therefore benefit from training prior to the use of the HD (3).

Since the development of the HD, data collected using this method have been used as a central endpoint of many clinical trials on PD (4), primarily due to its usefulness during long-term follow-up and limited clinician bias. However, despite widespread use, the HD assessments have not been compared to what is considered the gold standard for objective measurement of motor function in PD: assessment by an experienced observer.

The aim of this study was thus to validate the HD by investigating the agreement between observer and HD ratings.

## Materials and Methods

### Study Protocol Approvals and Patient Consents

This observational study was conducted at the Neurology Research Unit, Skåne University Hospital, Lund, Sweden (“the site”) as part of an international collaboration on symptom fluctuations in PD, VALIDATE-PD. The study was approved by the Regional Ethics Review Board, Lund, Sweden (2017/936) and informed written consent was obtained from study participants.

### Participants

Participants were recruited at the Department of Neurology, Skåne University Hospital or through the Swedish Parkinson Registry. Potential participants received information about the study in the mail and were then contacted by phone. Potential participants were invited to a screening visit that included the signing of an informed consent, evaluation of participation criteria, and documentation of baseline demographic and clinical information.

The inclusion criteria were: diagnosis of PD according to the United Kingdom PD Society Brain Bank criteria, age ≥30 years, motor fluctuations documented in patient records and/or on the revised Unified Parkinson's Disease Rating Scale (MDS-UPDRS), and the ability to sign an informed consent.

The exclusion criteria were: signs of secondary/atypical Parkinsonian syndromes, inability to complete patient questionnaires, lack of cooperation during study, signs of dementia [Montreal Cognitive Assessment (MoCA) ≤21] (5) or psychotic symptoms, and current device-aided treatment, as well as conditions interfering with the patient's ability to consent, adherence to the study protocol, or clinical evaluation.

### Instruments and Assessments

The MoCA was used for screening for cognitive impairment (maximum 30 points, lower scores indicate more cognitive impairment) (6). The MDS-UPDRS was used for characterization of the study sample (maximum 260 points, higher scores indicate more PD symptoms) (7).

The motor states that were selectable for observers and in the HD were identical: “Asleep”, “Off”, “On without dyskinesia”, “On with non-troublesome dyskinesia”, and “On with troublesome dyskinesia”. “On with dyskinesia” replaced the latter two categories in the analyses unless otherwise noted.

### Procedures

Each participant attended one screening visit on-site, completed one day of HD recording at home, and then two office-hour days on-site. Participants were instructed in the use of a HD and received oral (~10 min) and written instructions including pictograms on the HD motor states. No instruction videos or concordance thresholds were used. Participants were asked to use the HD for 24 h while at home and were then allowed to clarify any issues regarding the rating procedure with study personnel before starting on-site ratings. Only the on-site ratings were used in the analyses.

During the two days on-site, participants were asked every 30 min between 8 am and 4 pm to rise from a chair, walk seven meters, and note their motor state in the HD. Meanwhile, the observer made a simultaneous assessment blinded to the HD rating. The observer assessment was based on observations during preparation for and execution of the seven-meter walk. Aggregated diary data consisted of percentage daily times spent in the three motor state calculated as the mean from the two on-site days. In between the half-hourly assessments, participants were typically socializing, solving crossword puzzles, playing cards, reading magazines, listening to radio, having lunch, and drinking coffee.

Authors JT, SC, and three research nurses functioned as observers and the median experience of working with clinical PD research was about 5 years. All observers had completed the MDS-UPDRS training program prior to the study.

### Statistical Analyses

The McNemar-Bowker test was used to test for symmetry of disagreements between the rating procedures, while Cohen's κ was used to estimate the agreement between the observer and HD data (8). The McNemar test with Bonferroni adjustment for multiple comparisons was performed as *post-hoc* comparisons of the different motor states. The McNemar test was used to compare dyskinesia occurrence and severity between observer and HD assessments. Pearson's correlation test and intraclass correlation coefficient (ICC) estimation were used for correlations of daily times spent in the various motor states on the participant level. A Pearson's correlation coefficient |r| <0.3 was considered a weak, |r| = 0.3–0.59 a moderate and |r|≥0.6 a strong agreement/correlation. ICC estimates and their 95% confidence intervals (95% CIs) were calculated based on single-rating, absolute-agreement, 2-way mixed-effects models with two rating instruments across all participants. According to the guideline by Cichetti (9), we interpreted κ values or ICC < 0.40 as poor, κ/ICC = 0.40–0.59 as fair, κ/ICC = 0.60–0.74 as good and κ/ICC = 0.75–1.00 as excellent reliability. The Wilcoxon signed-rank test was used for ancillary comparison of the estimations from the MDS-UPDRS of time spent in “Off” and “On with dyskinesia” to the observer and HD assessments. The effect size of the Wilcoxon signed-rank test was calculated using $r\;=\;\frac{Z}{\sqrt{N}}$. *P* < 0.05 was considered significant. IBM SPSS Statistics version 26.0 was used for statistical analyses.

## Results

### Cohort Characteristics

Eighty-one potential participants received written information about the study and 41 (50.6%) agreed to participate. One participant declined further participation due to undisclosed reasons after the screening visit, while 40 participants completed the study (for demographic and clinical characteristics, see Table 1). No participant failed to comply with diary ratings.

**Table 1:** Demographic data, disease characteristics, and clinical instruments (*n* = 40).

Male/female	22 (55%)/18 (45%)
Age, in years, median (IQR)	70 (62–76)
Disease duration, in years, median (IQR)	7 (6–12)
Symptom duration, in years, median (IQR)	10 (7–14)
Duration of motor fluctuations, in months, median (IQR)	51 (25–79)
Hypokinetic fluctuations	46 (20–74)
Hyperkinetic fluctuations	36 (21–59)
MDS-UPDRS total, median (IQR)	45 (30–57)
Part I	8 (5–11)
Part II	8 (5–14)
Part III	20 (15–29)
Part IV	5 (3–8)
Hoehn & Yahr stage, median (IQR)	2 (2–3)
Motor fluctuation symptoms	
Nightly “off”	31 (78%)
“Wearing off”	30 (75%)
Delayed “on” or no “on”	9 (23%)
“On-off” phenomena	25 (64%)
Peak dose dyskinesia	27 (69%)
Biphasic dyskinesia	5 (14%)
Off-dose dystonia	12 (33%)
MoCA total	26 (24–28)
Cognitive Impairment	
Normal	22 (55%)
Mild Cognitive Impairment	18 (45%)
Dementia	0 (0%)
Antiparkinson medication	
Levodopa	40 (100%)
Catechol-*O*-methlytransferase (COMT) inhibitors	24 (60%)
Monoaminoxidase B (MAO-B) inhibitors	26 (65%)
Dopamine agonists	32 (80%)
Levodopa dose per day in mg, median (IQR)	525 (456–769)
Levodopa equivalent dose per day in mg, median (IQR)	941 (763–1187)

*Values are provided as numbers (percentages) or median with interquartile range (IQR); Mild cognitive impairment was defined as having a MoCA score of 22–25 points. MoCA, Montreal Cognitive Assessment; MDS-UPDRS, revised Unified Parkinson's Disease Rating Scale. Levodopa equivalent doses were calculated according to Tomlinson et al. (10)*.

### Comparisons of Observer Ratings and HD on the Half-Hour Level

**Figure 1:**
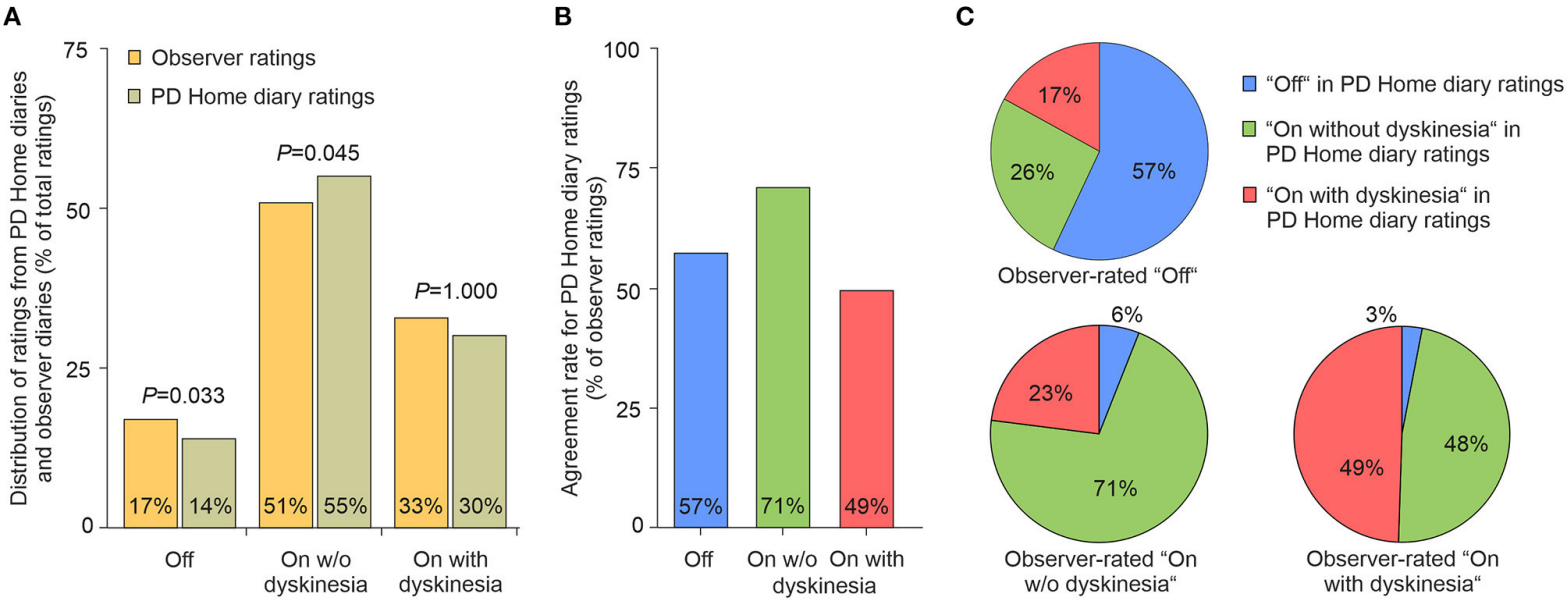
Proportion and temporal agreement of motor states assessed by observer diaries and PD Home diaries. **(A)** Proportion of “Off”, “On without dyskinesia” and “On with dyskinesia” as assessed by observer and patient diaries. *P*-values are from *post-hoc* McNemar test with Bonferroni adjustment. **(B)** Temporal agreement rate for all motor states as well as “Off”, “On without dyskinesia” and “On with dyskinesia” with the observer ratings as the reference for comparison. **(C)** Participants' choices on the PD Home diary in each respective observed motor state. Number of observations in each observed motor state: “Off”: *n* = 218, “On without dyskinesia”: *n* = 651, “On with dyskinesia”: *n* = 419. PD, Parkinson's disease.

Out of 2,720 expected half-hour ratings, 89 (3.3%) were missing. A total of 1,322 observer and 1,309 patient diary ratings resulted in 1,288 complete pairs of ratings. As displayed in Figure 1A, ratings in observer diaries and PD Home diaries were distributed between “Off”, “On without dyskinesia” and “On with dyskinesia” with a significant difference between observers and patient diary ratings in the distribution between the different motor states (*P* < 0.001), which was also illustrated by a Cohen's κ of 0.358. *Post-hoc* analyses comparing the various motor states revealed significant differences between the two ratings for “Off” (*P* = 0.033; McNemar test with Bonferroni adjustment) with a corresponding Cohen's κ of 0.562 and for “On without dyskinesia” (*P* = 0.045; McNemar test with Bonferroni adjustment) with a corresponding Cohen's κ of 0.314. Although there was no significant difference in the number of dyskinesia ratings between observers and HDs independent of their “troublesomeness” (Figure 1A, *P* = 1.000; Cohen's κ of 0.289), dyskinesia was significantly less often seen as “troublesome” in observer (2.1%) than patient diary ratings (10.9%, *P* < 0.001).

The agreement between observers and participants, using the observer ratings as the gold standard, ranged from 71.1% in “On without dyskinesia” to 49.4% in “On with dyskinesia” (Figure 1B). Patients considered themselves to be “On without dyskinesia” in 25.7% of the intervals with observed Off (Figure 1C). Even more strikingly, patients chose “On without dyskinesia” in 47.3% of those intervals in which the observer had actually noted “On with dyskinesia”.

### Comparisons of Observer Ratings and HD on the Participant Level

**Figure 2:**
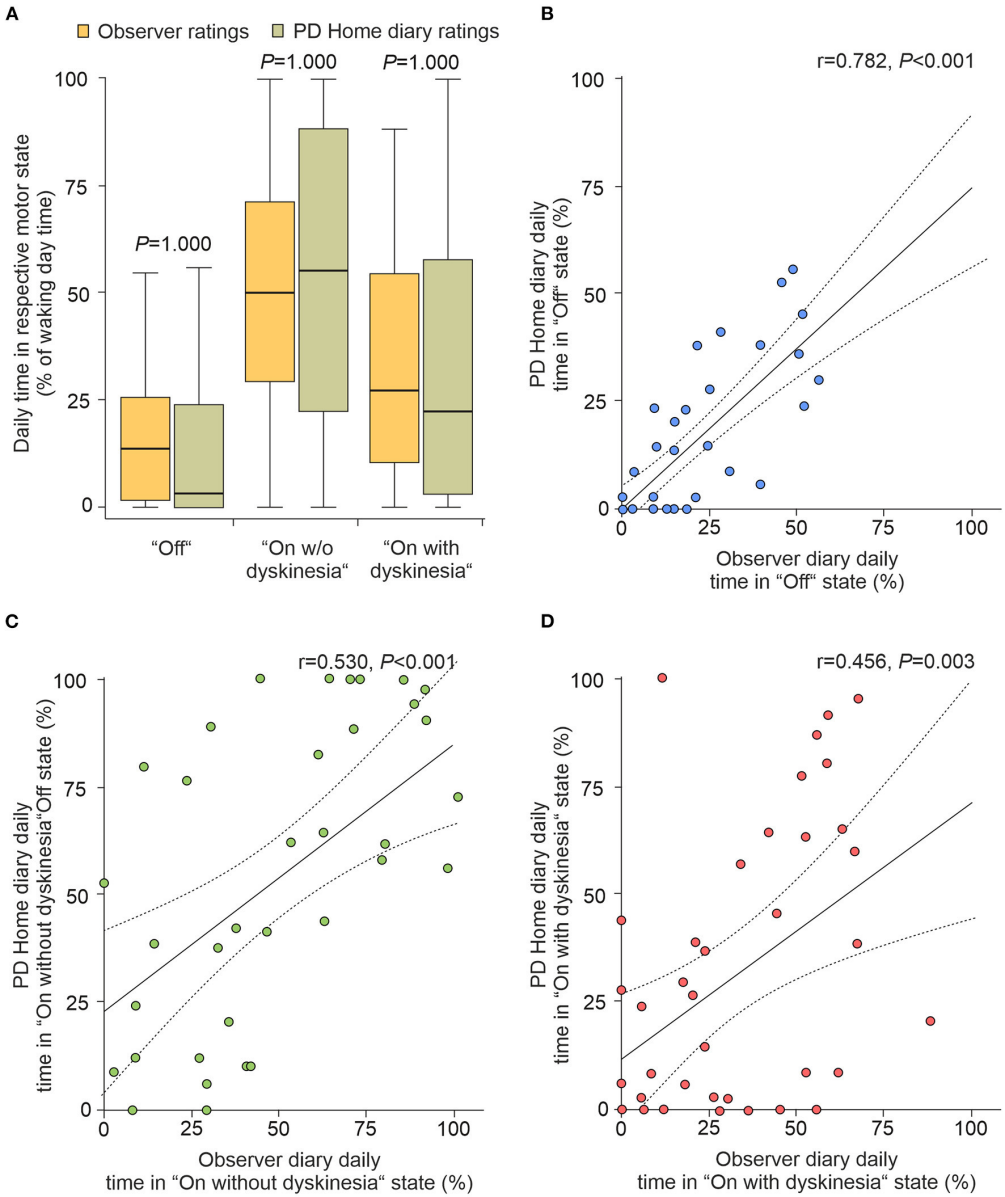
Proportions for time spent in motor states assessed by observer diaries and PD Home diaries on participant level. **(A)** Distribution of daily time proportions of “Off”, “On without dyskinesia” and “On with dyskinesia” based on the simultaneous, half-hourly performed diary ratings from 40 participants from two consecutive days (8 am to 4 pm). Boxplots are shown with a central mark at the median, bottom, and top edges of the boxes at 25th and 75th percentiles, respectively, whiskers out to the most extreme points within 1.5 times the interquartile range. Displayed *P*-values are from Friedmann tests with *post-hoc* Wilcoxon signed-rank tests with Bonferroni correction for multiple comparisons. **(B–D)** Correlation analyses of mean proportions of “Off” **(B)**, “On without dyskinesia” **(C)** and “On with dyskinesia” **(D)**. Solid lines in represent the regression line with 95% CI (dotted lines). Values in upper right corner are the correlation coefficients and *P-*values from Pearson's correlation tests. PD, Parkinson's disease; CI, confidence interval.

The HD have been repeatedly used as the primary outcome measure to assess effects of novel treatments on motor fluctuations in advanced PD with the aggregates measure of daily times spent in the three different motor states as the most frequent read-outs (4). We therefore also analyzed the daily percentage times spent in the three different motor states (8 am to 4 pm) on the participant level from all 40 participants. As shown in Figure 2A, we detected similar percentage daily times spent in all three motor states when comparing observer diary data and HD with no significant differences between the two diary ratings for all motor states (*P* ≥ 0.05, Friedman test with *post-hoc* Wilcoxon Rank test with Bonferroni adjustment). Pearson correlation analyses of the individual times spent in the three different motor states revealed a strong correlation of percentage daily times spent in “Off”, but only a moderate correlation of “On without dyskinesia” and “On with dyskinesia” between observer and patient diary data (Figures 2B–D). Reliability analyses using ICC calculation revealed excellent reliability for HD data for “Off” when correlated with observer diary data [ICC = 0.77 (95% CI: 0.60–0.87)], and fair reliability for “On without dyskinesia” [ICC = 0.52 (95% CI: 0.26–0.72)] and “On with dyskinesia” [ICC = 0.45 (95% CI: 0.16–0.67)], respectively.

Using the participants' estimation of waking hours spent in “On with dyskinesia” from the MDS-UPDRS item 4.1 for ancillary analyses, dyskinesia was found to be underreported in the MDS-UPDRS (median 12.5%) when compared to observer (median 27.9%, *P* < 0.001, *r* = −0.43) and HD (median 22.4%, *P* < 0.013, *r* = −0.28). There were no significant differences between the estimation of time spent in “Off” in the MDS-UPDRS item 4.3 (median 6.7%) and neither observer assessment (median 13.6%, *P* = 0.066, *r* = −0.21) nor HD ratings (median 3.4%, *P* = 0.852, *r* = −0.02).

**Figure 3:**
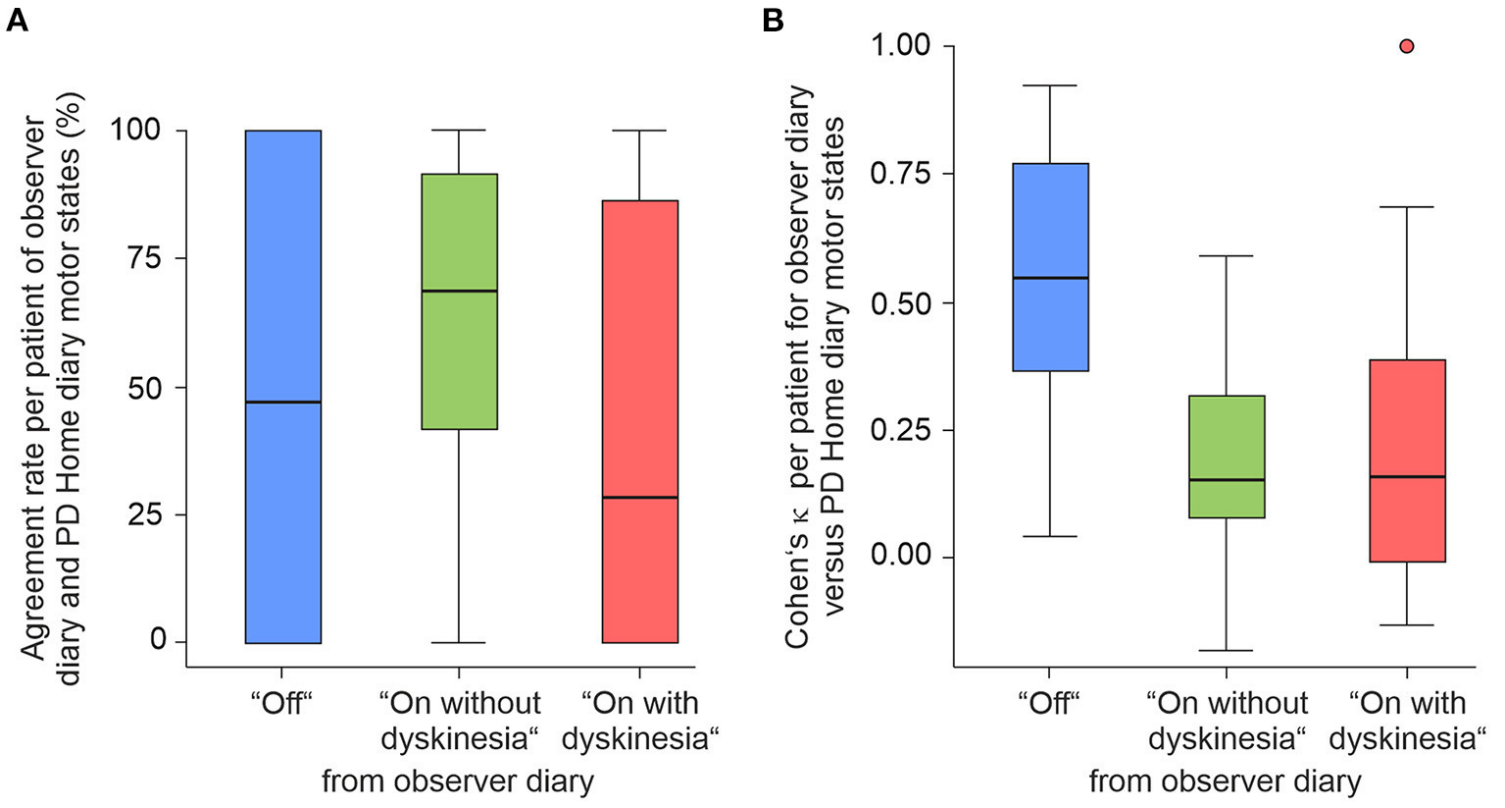
Temporal agreement of observer-documented and PD Home diary ratings on the participant level. Agreement rates **(A)** and Cohen's κ values **(B)** for “Off”, “On without dyskinesia” and “On with dyskinesia” based on simultaneous half-hourly ratings of 40 participants from two consecutive days (8 am to 4 pm) and independent observer ratings serving as reference for the comparison. Boxplots are shown with a central mark at the median, bottom, and top edges of the boxes at 25th and 75th percentiles, respectively, whiskers out to the most extreme points within 1.5 times the interquartile range, and outliers scoring more than 1.5 × IQR but at most 3 × IQR outside the quartiles. PD, Parkinson's disease; IQR, interquartile range.

The temporal agreement on the participant level of HD data with the observer-rated diary data were estimated using the temporal agreement rate and Cohen's κ for each participant (Figures 3A,B). Taking the observer diary as gold standard criterion, temporal agreement rates for HD data showed a very high variability within the cohort with median agreement rates of 56.3% of observer-rated “Off”, 68.8% of “On without dyskinesia”, and 28.3% of “On with dyskinesia” (Figure 3A). The corresponding median Cohen's κ values ranged from 0.15 for “On without dyskinesia” and “On with dyskinesia” to 0.55 for “Off” (Figure 3B).

## Discussion

The primary finding of this study is that the temporal agreement between simultaneous observer and HD assessments of the participants' PD motor state can be characterized as poor. Indeed, we found that as few as 49% of HD ratings in observed “On with dyskinesia” and 57% in observed “Off” were in agreement with the simultaneous observer assessment, while 71% of ratings in observed “On without dyskinesia” were in agreement. Analyses of temporal agreement on the participant levels resulted in very high variability of agreement rates between the participants, but in general similar results as on the time level. In contrast, for daily time spent in the three motor states as a major outcome measure in clinical studies (4), the HD show fair reliability for both “On” either with or without dyskinesia and even an excellent reliability for “Off” when using the observer diary data as the outside criterion.

As the HD and observer data was nominal, Cohen's κ was chosen over other possible methods for studying validity. The poor agreement between observers and participants (κ = 0.358) indicates that there were conflicting assessments of the participant's motor state (9). Participants were most successful at recognizing “On without dyskinesia” and least successful at recognizing “On with dyskinesia”. Participants and observers were largely in agreement regarding when the participant was in “On” if the severity of dyskinesia was not taken into account, but in observed “Off” 42.7% of the HD ratings were instead “On” either with or without dyskinesia (Figure 1C). The motor state is likely to overlap with other symptoms that are not noticeable to an observer but nonetheless make up a significant part of the patient's experience. This has historically led to difficulties with establishing a widely used practical definition of “Off” (11). Fluctuations of neuropsychiatric, sensory, and autonomic symptoms are generally present among PD patients with motor fluctuations (12). It is possible that such non-motor fluctuations could have influenced HD ratings.

Patients often prefer dyskinesia to hypokinesia (13) and the clinical impression is that observers are more likely to notice mild dyskinesia than patients themselves are. We did not find any significant difference in the number of “on with dyskinesia” ratings between HD and observer (*P* = 0.192) and cannot, based on our findings, support that notion. Instead, we show that participants rated dyskinesia as “troublesome” more often than observers did (*P* < 0.001). We refrained from further analysis regarding dyskinesia severity as it is an inherently subjective dichotomization. It is noteworthy that in observed “Off”, “On with dyskinesia” made up 17% of HD ratings, which may indicate a lack of understanding among participants of the PD motor states' characteristics, such as confusing tremor with dyskinesia.

Daily times spent in the three different motor states calculated from the HD have been repeatedly used as the primary outcome measures to assess effects of novel treatments on motor fluctuations in advanced PD (4). In reasonable agreement with Löhle et al. (14), the aggregated HD data showed fair to excellent reliability with ICC values ranging from 0.45 for “On with dyskinesia” over 0.52 for “On without dyskinesia” to 0.77 for “Off”. This rather good reliability of the aggregated data stand in contrast to the limited temporal agreement between HD and observer ratings. It is likely that the timing of motor and non-motor fluctuations in conjunction with their ratings limit the temporal agreement together with the differences in motor state perception between the patient and the objective observer (15). During further ancillary analyses, we found dyskinesia to be underreported in the MDS-UPDRS item 4.1, but the time spent in “Off” estimated in MDS-UPDRS item 4.3 did not significantly differ from neither the observer nor HD ratings. Although this is an interesting exploratory finding, our on-site ratings did not include the night-time, during which especially “Off” is common, and further investigation is warranted.

This study has several limitations. Firstly, all observers had experience of movement disorders and were certified in the use of MDS-UPDRS, but were not Movement Disorder Specialists and could thus be considered less accurate than the gold standard. Furthermore, using multiple observers may have influenced the results and, as each patient was rated by a single observer, no calculations of the inter-rater reliability between observers were performed (e.g., the Fleiss' kappa). However, findings from a single-rater German cohort are in many aspects in agreement with the present results (14). It is also possible that participants were more inactive than they would have been in a home setting and therefore were less likely to notice “Off” and troublesome dyskinesia due to a limited number of activities available at the site. Lastly, the participant instructions for the use of the HD could have been more rigorous and included the recommended instruction video (3), which might have increased the agreement with observer assessments. However, the level of instructions to participants in this study was representative of how the HD is often used in clinical trials and in clinical practice.

The Movement Disorders Society Technology Task Force has identified a number of limitations among the currently available PD patient diaries and proposed a comprehensive development plan for a new eDiary (16). The Task Force has for example highlighted the need for capturing partial medication states, medication intake, non-motor fluctuations, and functional assessments in the eDiary to better reflect the dynamic PD symptomatology. The eDiary is therefore intended to be an electronic diary/tracker interface that puts together the complementary information from patient ratings and wearable sensors.

The eDiary is certainly warranted, but the HD is likely to serve as a mainstay in clinical trials for several years to come. Based on our findings, and if observer assessments are held as the gold standard, the HD does not seem to be an accurate depiction of a patient's motor state at a given time point. However, that does not imply that the HD is not a useful tool since the daily time spent in the various motor states seems to reflect the observer times in a reliable manner. The HD should still be regarded as an important patient reported outcome, albeit a composite that is likely to be influenced by timing and other factors of the patient's experience than strictly the observed motor state. There is a potential complementary role for wearable sensors and other technology-based objective measures in the monitoring of PD, but it needs further study and we want to highlight the need for validation against observer ratings before implementation. Furthermore, it is warranted to investigate the effect of more extensive patient training on the agreement between HD and observer ratings. Notably, the limited temporal agreement might be particularly relevant in standard clinical use of the HD, wherein it is routinely applied to adapt the timing of antiparkinsonian medication.

## Data Availability Statement

The raw data supporting the conclusions of this article will be made available by the authors upon reasonable request that is in line with local data protection regulations.

## Ethics Statement

The studies involving human participants were reviewed and approved by the Regional Ethics Review Board, Lund, Sweden. The patients/participants provided their written informed consent to participate in this study.

## Author Contributions

JT, ML, AB, AS, and PO: conception and organization of research project, design and execution of statistical analysis, and review and critique of manuscript. JT, ML, AB, and SC: execution of research project. SC, FG, GE, ÖD, SI, and MN: review and critique of statistical analysis and review and critique of manuscript. JT: writing of the first draft. All authors contributed to the article and approved the submitted version.

## Funding

The authors declare that this study received funding from Global Kinetics Corporation, Melbourne, Australia and the Skåne University Hospital Foundation and Donations. JT received funding from the Swedish National Government and County Councils through the ALF agreement. The funders were not involved in the study design, collection, analysis, interpretation of data, the writing of this article, and the decision to submit it for publication.

## Conflict of Interest

JT has received funding from the Swedish National Government and County Councils through the ALF agreement, the Swedish Parkinson Foundation, and the Elsa Schmitz Foundation, and has received compensation for consultancies from AbbVie and TransPerfect, as well as royalties from UNI-MED Verlag. ML has received honoraria for presentations from Novartis Pharma and UCB Pharma. FG reports honoraria from AbbVie, BIAL, Merz, and STADA outside the submitted work. GE received honoraria for Advisory Boards, Consultancy and Presentations from AbbVie Pharma, BIAL Pharma, Biogen GmbH, Desitin Pharma, STADA Pharma, Neuroderm Inc. Licher GmbH, UCB Pharma, Zambon Pharma. He has received royalties from Kohlhammer Verlag and Thieme Verlag. ÖD has received funding from Faculty of Arts and Sciences, Linköping University. SI has received honoraria from Karolinska Institutet and Swedish Agency for Participation. She has received research funding from Ribbingska Foundation in Lund, Swedish Research Council; Swedish Research Council for Health, Working Life and Welfare (Forte); Swedish Research Council for Sustainable Development (Formas), NEURO Sweden, Family Kamprad Foundation, LMK Foundation, Lund University, Lund University Innovation System, Helsingborg City Competence Foundation. MN was funded by the Strategic Research Area in neuroscience (MultiPark) at Lund University, Lund, Sweden. AS has received funding from the Deutsche Forschungsgemeinschaft (German Research Association) and the Helmholtz-Association. He has received honoraria for presentations/advisory boards/consultations from Desitin, Global Kinetics, Lobsor Pharmaceuticals, STADA, Bial, RG Gesellschaft, Zambon, and AbbVie. He has received royalties from Kohlhammer Verlag and Elsevier Press. He serves as an editorial board member of Stem Cells and Stem Cells International. PO has received funding from AbbVie, Lund University Medical Faculty, Multipark, the Swedish Parkinson Foundation, Health Care Region Skåne and Åhlens Foundation. He has received honoraria for lectures and expert advice from AbbVie, Bial, Britannia, Ever Pharma, Global Kinetics, Lobsor, Nordic Infucare, Stada, and Zambon. He has received royalties from UNI-MED Verlag. The remaining authors declare that the research was conducted in the absence of any commercial or financial relationships that could be construed as a potential conflict of interest.

## Publisher's Note

All claims expressed in this article are solely those of the authors and do not necessarily represent those of their affiliated organizations, or those of the publisher, the editors and the reviewers. Any product that may be evaluated in this article, or claim that may be made by its manufacturer, is not guaranteed or endorsed by the publisher.
